# (*E*)-*N*′-(2-Thienyl­methyl­idene)-*p*-toluene­sulfono­hydrazide

**DOI:** 10.1107/S1600536810032708

**Published:** 2010-08-21

**Authors:** Abdullah M. Asiri, Mohie E. M. Zayed, Seik Weng Ng

**Affiliations:** a Chemistry Department, Faculty of Science, King Abdul Aziz University, PO Box 80203, Jeddah 21589, Saudi Arabia; b Department of Chemistry, University of Malaya, 50603 Kuala Lumpur, Malaysia

## Abstract

The S—N(H)—N=C linkage in the title mol­ecule, C_12_H_12_N_2_O_2_S_2_, is non-planar [torsion angle = 15.5 (1)°] as the amino N atom is pyramidally coordinated. The amino group acts as a hydrogen-bond donor to an O atom of an adjacent mol­ecule, generating chains running parallel to the *c* axis.

## Related literature

For the structure of the (*E*)-*N*′-benzyl­idene-*p*-toluene­sulf­ono­hydrazide homolog, see: Mehrabi *et al.* (2008 ▶).
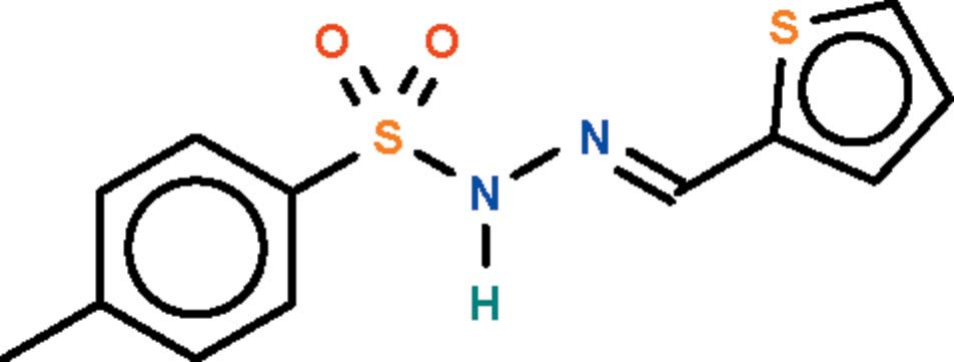


## Experimental

### 

#### Crystal data

C_12_H_12_N_2_O_2_S_2_
*M* *_r_* = 280.36
Monoclinic, 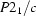

*a* = 14.3758 (10) Å
*b* = 9.8613 (7) Å
*c* = 9.6172 (7) Å
β = 104.981 (1)°
*V* = 1317.03 (16) Å^3^
*Z* = 4
Mo *K*α radiation
μ = 0.40 mm^−1^
*T* = 100 K
0.40 × 0.20 × 0.20 mm

#### Data collection

Bruker SMART APEX diffractometer
Absorption correction: multi-scan (*SADABS*; Sheldrick, 1996 ▶) *T*_min_ = 0.857, *T*_max_ = 0.925
8238 measured reflections
3022 independent reflections
2728 reflections with *I* > 2σ(*I*)
*R*_int_ = 0.020

#### Refinement

*R*[*F* ^2^ > 2σ(*F* ^2^)] = 0.030
*wR*(*F* ^2^) = 0.085
*S* = 1.04
3022 reflections
168 parameters
1 restraint
H atoms treated by a mixture of independent and constrained refinement
Δρ_max_ = 0.42 e Å^−3^
Δρ_min_ = −0.36 e Å^−3^

### 

Data collection: *APEX2* (Bruker, 2009 ▶); cell refinement: *SAINT* (Bruker, 2009 ▶); data reduction: *SAINT*; program(s) used to solve structure: *SHELXS97* (Sheldrick, 2008 ▶); program(s) used to refine structure: *SHELXL97* (Sheldrick, 2008 ▶); molecular graphics: *X-SEED* (Barbour, 2001 ▶); software used to prepare material for publication: *publCIF* (Westrip, 2010 ▶).

## Supplementary Material

Crystal structure: contains datablocks global, I. DOI: 10.1107/S1600536810032708/pk2261sup1.cif

Structure factors: contains datablocks I. DOI: 10.1107/S1600536810032708/pk2261Isup2.hkl

Additional supplementary materials:  crystallographic information; 3D view; checkCIF report

## Figures and Tables

**Table 1:** Hydrogen-bond geometry (Å, °)

*D*—H⋯*A*	*D*—H	H⋯*A*	*D*⋯*A*	*D*—H⋯*A*
N1—H1⋯O1^i^	0.86 (1)	2.06 (1)	2.874 (2)	159 (2)

Symmetry code: (i) 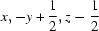.

**Fig. 1.**
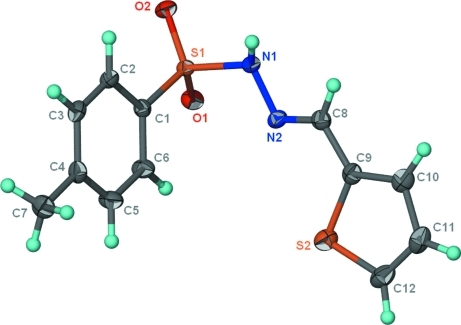
Thermal ellipsoid plot (Barbour, 2001) of C12H12N2O2S2 at the 70% probability level; hydrogen atoms are drawn as spheres of arbitrary radius.

**Fig. 2.**
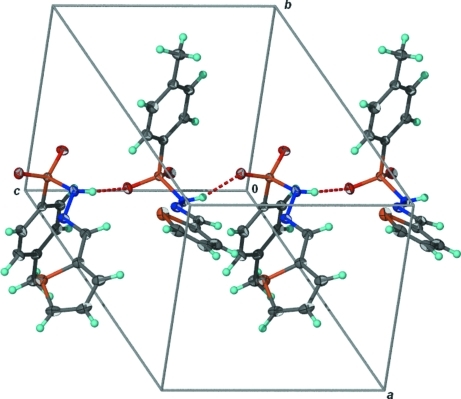
A view of the chain structure resulting from N—H···O hydrogen-bonding.

## supplementary crystallographic information

### Comment

*p*-Toluenesulfonyl hydrazide, CH_3_-4-C_6_H_4_SO_2_NHNH_2_, condenses with carbonyl compounds to form Schiff bases, and among the plethora nearly a hundred have had their crystal structures determined.  The compounds have the azomethine double-bond in an *E*-configuration. In the Schiff base product between *p*-toluenesulfonyl hydrazide and thiophene-2-carboxaldehyde, the S–N(H)–N═C linkage is non-planar [torsion angle 15.5 (1) °] because the amino nitrogen atom (which bears a hydrogen atom) is pyramidally coordinated (Fig. 1).  The amino group acts as a hydrogen-bond donor to an oxygen atom of an adjacent molecule to generate a chain running along the *c*-axis of the monoclinic cell (Fig. 2). The oxygen atom involved in hydrogen bonding [S–O 1.4355 (10) Å] is marginally farther from the sulfur atom than the oxygen that is not involved in hydrogen bonding [S–O 1.4288 (10) Å].

### Experimental

*p*-Toluenesulfonyl hydrazide (4.66 g, 2.5 mmol) and thiophene-2-carboxaldehyde (2.80 g, 2.5 mmol) were heated in methanol (50 ml) for two hours. The cool solution yielded a precipitate that was recrystallized from ethanol and collected in 90% yield.

### Refinement

Carbon-bound H-atoms were placed in calculated positions [C–H 0.95 to 0.99 Å, *U*(H) 1.2 to 1.5*U*_eq_(C)] and were included in the refinement in the riding model approximation.  The amino H-atom was located in a difference Fourier map, and was refined with a distance restraint [N–H 0.86 (1) Å]; its temperature factor was freely refined.

### Crystal data

**Table:**

C_12_H_12_N_2_O_2_S_2_	*F*(000) = 584
*M**_r_* = 280.36	*D*_x_ = 1.414 Mg m^−^^3^
Monoclinic, *P*2_1_/*c*	Mo *K*α radiation, λ = 0.71073 Å
Hall symbol: -P 2ybc	Cell parameters from 4750 reflections
*a* = 14.3758 (10) Å	θ = 2.5–28.3°
*b* = 9.8613 (7) Å	µ = 0.40 mm^−^^1^
*c* = 9.6172 (7) Å	*T* = 100 K
β = 104.981 (1)°	Prism, yellow
*V* = 1317.03 (16) Å^3^	0.40 × 0.20 × 0.20 mm
*Z* = 4	

### Data collection

**Table:**

Bruker SMART APEX diffractometer	3022 independent reflections
Radiation source: fine-focus sealed tube	2728 reflections with *I* > 2σ(*I*)
graphite	*R*_int_ = 0.020
ω scans	θ_max_ = 27.5°, θ_min_ = 2.5°
Absorption correction: multi-scan (*SADABS*; Sheldrick, 1996)	*h* = −18→18
*T*_min_ = 0.857, *T*_max_ = 0.925	*k* = −12→12
8238 measured reflections	*l* = −8→12

### Refinement

**Table:**

Refinement on *F*^2^	Primary atom site location: structure-invariant direct methods
Least-squares matrix: full	Secondary atom site location: difference Fourier map
*R*[*F*^2^ > 2σ(*F*^2^)] = 0.030	Hydrogen site location: inferred from neighbouring sites
*wR*(*F*^2^) = 0.085	H atoms treated by a mixture of independent and constrained refinement
*S* = 1.04	*w* = 1/[σ^2^(*F*_o_^2^) + (0.0458*P*)^2^ + 0.7746*P*] where *P* = (*F*_o_^2^ + 2*F*_c_^2^)/3
3022 reflections	(Δ/σ)_max_ = 0.001
168 parameters	Δρ_max_ = 0.42 e Å^−^^3^
1 restraint	Δρ_min_ = −0.36 e Å^−^^3^

### Fractional atomic coordinates and isotropic or equivalent isotropic displacement parameters (Å^2^)

**Table:**

	*x*	*y*	*z*	*U*_iso_*/*U*_eq_	
S1	0.16267 (2)	0.25347 (3)	0.52894 (3)	0.01262 (10)	
S2	0.51633 (3)	0.43030 (4)	0.75091 (4)	0.02186 (11)	
O1	0.18378 (8)	0.21042 (11)	0.67638 (11)	0.0176 (2)	
O2	0.08674 (7)	0.18970 (10)	0.42362 (11)	0.0174 (2)	
N1	0.26105 (9)	0.22234 (13)	0.47478 (13)	0.0148 (2)	
H1	0.2522 (14)	0.235 (2)	0.3840 (11)	0.024 (5)*	
N2	0.34437 (9)	0.28097 (12)	0.56466 (13)	0.0156 (2)	
C1	0.14605 (10)	0.42992 (14)	0.52115 (15)	0.0136 (3)	
C2	0.08653 (10)	0.48722 (15)	0.39742 (15)	0.0159 (3)	
H2	0.0530	0.4313	0.3203	0.019*	
C3	0.07698 (10)	0.62763 (15)	0.38847 (15)	0.0170 (3)	
H3	0.0360	0.6673	0.3048	0.020*	
C4	0.12658 (10)	0.71101 (15)	0.50031 (16)	0.0172 (3)	
C5	0.18520 (11)	0.65074 (15)	0.62312 (16)	0.0206 (3)	
H5	0.2189	0.7065	0.7003	0.025*	
C6	0.19530 (11)	0.51082 (15)	0.63480 (15)	0.0191 (3)	
H6	0.2353	0.4710	0.7192	0.023*	
C7	0.11737 (12)	0.86307 (15)	0.48857 (17)	0.0216 (3)	
H7A	0.1290	0.9025	0.5851	0.032*	
H7B	0.0524	0.8870	0.4322	0.032*	
H7C	0.1647	0.8987	0.4408	0.032*	
C8	0.41651 (11)	0.28677 (14)	0.51016 (16)	0.0169 (3)	
H8	0.4109	0.2523	0.4161	0.020*	
C9	0.50662 (10)	0.34545 (15)	0.59069 (16)	0.0173 (3)	
C10	0.59275 (11)	0.34852 (15)	0.55085 (17)	0.0208 (3)	
H10	0.6019	0.3079	0.4658	0.025*	
C11	0.66556 (11)	0.41995 (16)	0.65279 (19)	0.0235 (3)	
H11	0.7291	0.4324	0.6431	0.028*	
C12	0.63483 (11)	0.46843 (16)	0.76529 (18)	0.0238 (3)	
H12	0.6745	0.5179	0.8432	0.029*	

### Atomic displacement parameters (Å^2^)

**Table:**

	*U*^11^	*U*^22^	*U*^33^	*U*^12^	*U*^13^	*U*^23^
S1	0.01349 (18)	0.01241 (17)	0.01124 (17)	−0.00057 (12)	0.00189 (13)	0.00002 (11)
S2	0.0198 (2)	0.0240 (2)	0.0209 (2)	−0.00308 (14)	0.00374 (15)	−0.00061 (14)
O1	0.0225 (5)	0.0173 (5)	0.0128 (5)	−0.0004 (4)	0.0045 (4)	0.0019 (4)
O2	0.0160 (5)	0.0164 (5)	0.0175 (5)	−0.0029 (4)	0.0002 (4)	−0.0010 (4)
N1	0.0137 (6)	0.0176 (6)	0.0120 (6)	0.0007 (4)	0.0014 (4)	−0.0019 (4)
N2	0.0142 (6)	0.0142 (5)	0.0163 (6)	−0.0001 (4)	0.0000 (5)	0.0006 (4)
C1	0.0136 (6)	0.0128 (6)	0.0149 (6)	0.0002 (5)	0.0048 (5)	0.0003 (5)
C2	0.0133 (6)	0.0180 (7)	0.0152 (6)	−0.0005 (5)	0.0018 (5)	−0.0011 (5)
C3	0.0136 (6)	0.0192 (7)	0.0168 (7)	0.0024 (5)	0.0017 (5)	0.0031 (5)
C4	0.0161 (7)	0.0159 (7)	0.0210 (7)	0.0011 (5)	0.0073 (6)	0.0017 (5)
C5	0.0249 (7)	0.0170 (7)	0.0177 (7)	−0.0016 (6)	0.0013 (6)	−0.0032 (6)
C6	0.0226 (7)	0.0179 (7)	0.0138 (7)	0.0015 (6)	−0.0005 (5)	0.0002 (5)
C7	0.0246 (8)	0.0143 (7)	0.0256 (8)	0.0008 (6)	0.0062 (6)	0.0017 (6)
C8	0.0184 (7)	0.0140 (6)	0.0176 (7)	0.0017 (5)	0.0032 (5)	0.0006 (5)
C9	0.0169 (7)	0.0145 (6)	0.0199 (7)	0.0012 (5)	0.0038 (5)	0.0026 (5)
C10	0.0203 (7)	0.0163 (7)	0.0248 (8)	−0.0010 (6)	0.0040 (6)	0.0040 (6)
C11	0.0164 (7)	0.0190 (7)	0.0340 (9)	−0.0011 (6)	0.0048 (6)	0.0077 (6)
C12	0.0189 (7)	0.0192 (7)	0.0293 (8)	−0.0042 (6)	−0.0006 (6)	0.0035 (6)

### Geometric parameters (Å, °)

**Table:**

S1—O2	1.4288 (10)	C4—C7	1.507 (2)
S1—O1	1.4355 (10)	C5—C6	1.389 (2)
S1—N1	1.6572 (13)	C5—H5	0.9500
S1—C1	1.7553 (14)	C6—H6	0.9500
S2—C12	1.7148 (16)	C7—H7A	0.9800
S2—C9	1.7270 (15)	C7—H7B	0.9800
N1—N2	1.4074 (16)	C7—H7C	0.9800
N1—H1	0.859 (9)	C8—C9	1.447 (2)
N2—C8	1.279 (2)	C8—H8	0.9500
C1—C6	1.3901 (19)	C9—C10	1.388 (2)
C1—C2	1.3932 (19)	C10—C11	1.422 (2)
C2—C3	1.392 (2)	C10—H10	0.9500
C2—H2	0.9500	C11—C12	1.357 (2)
C3—C4	1.395 (2)	C11—H11	0.9500
C3—H3	0.9500	C12—H12	0.9500
C4—C5	1.394 (2)		
			
O2—S1—O1	119.85 (6)	C4—C5—H5	119.3
O2—S1—N1	104.68 (6)	C1—C6—C5	119.03 (13)
O1—S1—N1	106.02 (6)	C1—C6—H6	120.5
O2—S1—C1	109.60 (6)	C5—C6—H6	120.5
O1—S1—C1	109.08 (7)	C4—C7—H7A	109.5
N1—S1—C1	106.74 (6)	C4—C7—H7B	109.5
C12—S2—C9	91.59 (8)	H7A—C7—H7B	109.5
N2—N1—S1	113.01 (9)	C4—C7—H7C	109.5
N2—N1—H1	116.3 (13)	H7A—C7—H7C	109.5
S1—N1—H1	112.1 (13)	H7B—C7—H7C	109.5
C8—N2—N1	114.73 (12)	N2—C8—C9	120.48 (14)
C6—C1—C2	120.95 (13)	N2—C8—H8	119.8
C6—C1—S1	119.95 (11)	C9—C8—H8	119.8
C2—C1—S1	119.03 (11)	C10—C9—C8	126.88 (14)
C1—C2—C3	119.03 (13)	C10—C9—S2	111.33 (11)
C1—C2—H2	120.5	C8—C9—S2	121.73 (11)
C3—C2—H2	120.5	C9—C10—C11	111.76 (14)
C4—C3—C2	121.08 (13)	C9—C10—H10	124.1
C4—C3—H3	119.5	C11—C10—H10	124.1
C2—C3—H3	119.5	C12—C11—C10	113.01 (14)
C3—C4—C5	118.60 (13)	C12—C11—H11	123.5
C3—C4—C7	120.76 (13)	C10—C11—H11	123.5
C5—C4—C7	120.65 (14)	C11—C12—S2	112.32 (12)
C6—C5—C4	121.31 (14)	C11—C12—H12	123.8
C6—C5—H5	119.3	S2—C12—H12	123.8
			
O2—S1—N1—N2	178.54 (9)	C3—C4—C5—C6	0.5 (2)
O1—S1—N1—N2	−53.83 (11)	C7—C4—C5—C6	−179.24 (15)
C1—S1—N1—N2	62.38 (11)	C2—C1—C6—C5	−0.6 (2)
S1—N1—N2—C8	−164.50 (11)	S1—C1—C6—C5	176.50 (12)
O2—S1—C1—C6	163.60 (12)	C4—C5—C6—C1	0.2 (2)
O1—S1—C1—C6	30.58 (14)	N1—N2—C8—C9	179.61 (12)
N1—S1—C1—C6	−83.57 (13)	N2—C8—C9—C10	174.24 (14)
O2—S1—C1—C2	−19.24 (14)	N2—C8—C9—S2	−9.0 (2)
O1—S1—C1—C2	−152.26 (11)	C12—S2—C9—C10	−0.45 (12)
N1—S1—C1—C2	93.59 (12)	C12—S2—C9—C8	−177.63 (13)
C6—C1—C2—C3	0.2 (2)	C8—C9—C10—C11	177.28 (14)
S1—C1—C2—C3	−176.95 (11)	S2—C9—C10—C11	0.27 (16)
C1—C2—C3—C4	0.6 (2)	C9—C10—C11—C12	0.12 (19)
C2—C3—C4—C5	−1.0 (2)	C10—C11—C12—S2	−0.46 (18)
C2—C3—C4—C7	178.80 (14)	C9—S2—C12—C11	0.52 (13)

### Hydrogen-bond geometry (Å, °)

**Table:**

*D*—H···*A*	*D*—H	H···*A*	*D*···*A*	*D*—H···*A*
N1—H1···O1^i^	0.86 (1)	2.06 (1)	2.874 (2)	159 (2)

Symmetry codes: (i) *x*, −*y*+1/2, *z*−1/2.
